# Decrease in preovulatory serum estradiol is a valuable marker for predicting premature ovulation in natural/unstimulated in vitro fertilization cycle

**DOI:** 10.1186/s13048-018-0469-x

**Published:** 2018-11-21

**Authors:** Xuefeng Lu, Shuzin Khor, Qianqian Zhu, Lihua Sun, Yun Wang, Qiuju Chen, Ling Wu, Yonglun Fu, Hui Tian, Qifeng Lyu, Renfei Cai, Yanping Kuang

**Affiliations:** 1 Department of Assisted Reproduction, Shanghai Ninth People’s Hospital, Shanghai Jiaotong University School of Medicine, 639 Zhizaoju Rd, Shanghai, 200011 China; 2 Department of Assisted Reproduction, Shanghai East Hospital, Shanghai Tongji University School of Medicine, Shanghai, 200120 China

**Keywords:** Preovulatory E2 decrease, Premature ovulation, Natural IVF cycle

## Abstract

**Background:**

Premature ovulation occurs at a high rate in natural-cycle in vitro fertilization (IVF), and cycle cancellation further hampers the overall efficiency of the procedure. While lower levels of estradiol (E2) are observed in preovulatory follicles, it is unclear whether declines in E2 can be used as an effective marker of premature ovulation.

**Methods:**

This retrospective analysis includes 801 natural/unstimulated IVF/ICSI cycles undergoing scheduled ovum pick-up (OPU) and 153 natural/unstimulated IVF/ICSI cycles undergoing emergency OPU at a university IVF center from May 2014 to February 2017.

**Results:**

Among the 801 IVF/ICSI cycles undergoing scheduled OPU, preovulatory E2 levels increased by more than 10% in 403 (50.31%) cycles of the sample (Group A), while 192 (23.97%) cycles experienced a plateau (increased or decreased by 10%; Group B), and 206 (25.72%) cycles decreased by more than 10% (Group C). Group C had more patients who experienced premature LH surges, premature ovulation, as well as the fewest oocytes retrieved, frozen embryos, and top-quality embryos. A multivariate logistic regression analysis indicated that premature ovulation was associated with preovulatory E2/−1E2 ratio and premature LH surge. Moreover, preovulatory E2/−1E2 ratio served as a valuable marker for differentiating premature ovulation, with an AUC (area under the receiver operating curve) of 0.708 and 0.772 in cycles with premature LH surges and cycles without premature LH surges, respectively. Emergency OPU resulted in a significantly decreased rate of premature ovulation and increased number of frozen embryos.

**Conclusion:**

Decreases in preovulatory serum E2 was a valuable marker for premature ovulation in natural/unstimulated IVF cycle. Emergency OPU based on the preovulatory E2/−1E2 ratio decreased the rate of premature ovulation in cycles that experienced E2 decreases.

**Electronic supplementary material:**

The online version of this article (10.1186/s13048-018-0469-x) contains supplementary material, which is available to authorized users.

## Introduction

Controlled ovarian hyperstimulation (COH) can enhance the efficiency of assistant reproduction treatment (ART) and is widely used [1]. Natural-cycle IVF is still a reasonable and efficient treatment alternative for both patients who respond normally and those who respond poorly [2, 3]. Natural-cycle IVF offers several advantages, including conferring no risk of ovarian hyperstimulation syndrome, lower costs, and the avoidance of stimulatory medications and physical ease, all of which allow cycles to be repeated more frequently [4–7]. Moreover, natural-cycle IVF could reduce adverse perinatal outcomes, such as low birth weight, in fresh embryo transfers performed following ovarian stimulation [8]. However, the efficiency of natural-cycle IVF is hampered by its high cancellation rate because of spontaneous luteinizing hormone (LH) surge and premature ovulation, which occur in more than 30% of initiated cycles [9, 10].

Various protocols have attempted to enhance the success of natural-cycle IVF [11, 12]. In unstimulated IVF, the oocyte is first monitored, and a retrieval date is planned from the moment the follicle approaches maturity (i.e., a follicle size of approximately 10 mm). LH levels are then monitored, and follicle aspiration is performed. Next, human chorionic gonadotropin (hCG) and GnRH agonists (GnRHa) are given prior to natural LH surges to facilitate more accurate scheduling of follicle aspiration. Following this procedure, premature ovulation continues to occur at a high rate, and cycle cancellation further hampers the overall efficiency of natural-cycle IVF [13]. A better marker capable of predicting premature ovulation could help avoid natural/unstimulated IVF cycle cancellation.

Oocyte maturity parallels both the progressive antral cavity enlargement and the production of E2 by granulosa cells. Several studies have found that E2 levels decrease on the day following hCG/GnRHa triggers and are associated with poor IVF outcomes [14]. Laufer et al. analyzed 144 women suffering from tubal factor infertility who underwent laparoscopic oocyte aspiration and found that preovulatory serum E2 decreases were associated with lower pregnancy rates. The authors advocated for cancelling retrieval in patients who displayed preovulatory serum E2 decreases [15]. Preovulatory E2 decreases were also found to result in impaired implantation [16]. However, few studies have assessed the prognostic value of decreases in preovulatory E2 as a predictor for premature ovulation.

In this study, we performed a large retrospective study and found that a preovulatory decrease in E2 was a useful marker for predicting premature ovulation and that immediate follicle aspiration can decrease natural cycle cancellation and increase natural cycle efficiency.

## Materials and method

### Study population and design

This is a retrospective study of patients attending the Department of Assisted Reproduction of the Ninth People’s Hospital of Shanghai Jiaotong University School of Medicine from May 2014 to February 2017. The criteria for study inclusion was that the patient be undergoing their first natural/unstimulated IVF cycle during the study period. The criteria for exclusion was that the patient did not have their E2 level measured after premature LH surges or hCG/GnRHa triggers. Diminished ovarian reserve (DOR) was defined by either a serum FSH value ≥11.4 mIU/ml on day 3 or a total antral follicle count ≤4 [17].

### Natural/unstimulated cycles

Natural/unstimulated cycles were performed, as shown in Additional file 1: Figure S1. Follicular size and serum FSH, LH, E2, and P were measured from the third day of the menstrual cycle, and no drug was used during the follicle development. When the dominant follicle diameter was ≥18 mm, had an E2 level ≥ 200 pg/ml, and an LH level < 20 mIU/ml, ovulation was triggered using either triptorelin (0.1 mg; Decapeptyl, Ferring Pharmaceuticals, Malmo, Sweden) or hCG 5000 IU (Lizhu Pharmaceutical Trading Co. Zhuhai, China) at 23:00. Transvaginal ultrasound–guided oocyte retrieval was conducted 32–34 h after the trigger was scheduled. Serum FSH, LH, E2, and P levels were measured 10 h later. If the patient’s E2 levels decreased, ovum pick-up (OPU) was performed either as scheduled or immediately, based on physician preference. If the LH levels were ≥ 20 mIU/ml (premature LH surge), serum FSH, LH, E2, and P levels were measured in the afternoon (six hours later), and OPU was performed at 8:00 in the next morning, as scheduled. If E2 levels decreased, OPU was performed either as scheduled or immediately. All follicles with diameters of more than 10 mm were aspirated. Fertilization of the aspirated oocytes was performed in vitro using either conventional insemination or intracytoplasmic sperm injection (ICSI), depending on the semen parameters. Embryos were examined for the number and regularity of blastomeres and the degree of embryonic fragmentation on the third day, according to criteria described by Cummins et al. [18]

There was a high ratio of natural cycles without embryos, and the embryos that developed were often unpredictable. Moreover, premature surges in LH and elevated P levels might interfere with the outcome of embryo transfers in the natural cycle. Therefore, the “freeze-all” strategy was performed for all IVF/ICSI cycles. All high-quality embryos, including grade І and grade II 8-cell embryos, were frozen via vitrification on the third day after oocyte retrieval. Only nonhigh-quality embryos were placed in extended culture until they reached the blastocyst stage. During this stage, only good-morphology blastocysts were frozen on day 5 or day 6. The vitrification procedure for freezing cleavage-stage embryos and blastocysts was performed as previously described [19].

For thawing, solutions of 1 M, 0.5 M, and 0 M sucrose were used sequentially as cryoprotectant dilutions. All vitrification and warming steps were performed at room temperature, except for the first warming step, which was conducted at 37 °C. Hormone replacement treatment was recommended for endometrial preparation. Briefly, ethinylestradiol 25 μg Tid (Xinyi Pharmaceutical Co., Shanghai, China) was administered for 14 days, and the treatment was then shifted to 4 Femoston (Abbott Healthcare Products, B.V., Weesp, Netherlands) yellow oral tablets daily (8 mg oestradiol and 40 mg dydrogesterone) and soft vaginal progesterone capsules (Utrogestan 200 mg Bid, Laboratoires Besins-Iscovesco, Paris, France). Embryo transfer was arranged after 3 days. Blastocyst transfers were performed after 5 days. Once pregnancy was achieved, exogenous estrogen and progesterone supplements were continued until 10 weeks of gestation.

### Hormone analysis

Serum FSH, LH, E2, and P levels were measured on MC3, the trigger day, the day after trigger (approximately 10 h after the injection of GnRHa / hCG), and six hours after LH ≥20 mIU/ml were observed. Hormone levels were measured using chemiluminescence (Abbott Biologicals B.V., Weesp, Netherlands). The lower limits of sensitivity used were 0.06 mIU/mL for FSH, 0.09 mIU/mL for LH, 10 pg/ mL for E2, and 0.1 ng/mL for P. The upper limit for E2 measurement was set at 5000 pg/mL, and levels were recorded as 5000 pg/mL if they were higher than the upper limit on the trigger day or the day after trigger.

### Statistical analysis

The oocyte retrieval rate was defined as the ratio of the total number of oocytes retrieved to the number of follicles with a diameter ≥ 10 mm aspirated on the day of oocyte retrieval. Oocyte maturity rate was defined as the ratio of MII oocytes to the number of collected oocytes. Live birth was defined as delivery of a live child after 28 weeks of gestation. Clinical pregnancy was defined as the presence of a gestational sac with fetal heart activity during ultrasound examination 7 weeks after FET. The implantation rate was defined as the number of gestational sacs divided by the number of transferred embryos.

For continuous variables, comparisons among groups were analyzed by ANOVA, followed by an appropriate post hoc test or t-test. For categorical variables, the Pearson’s Chi-square test or Fisher’s exact test was used to examine differences. *P*-values less than 0.05 were considered statistically significant. A logistic regression model was used to quantify the effect of related factors on the premature ovulation. The following factors were considered: female age, basal FSH level, trigger type (premature LH surge or hCG/GnRHa trigger), and preovulatory E2/−1E2 ratio. All factors were introduced into the regression model. The data in the table are presented as the mean and 95% confidence intervals (CI) of the mean. Receiver operating curve (ROC) analyses were used to assess and compare the predictive accuracy of the ratio (preovulatory E2/− 1E2). The optimal cut-off value for the ratio of E2 change was determined using the value that maximizes the measure of sensitivity + specificity − 1. ROC curves and all other data were analyzed using the Statistical Package for the Social Sciences for Windows (version 22, SPSS Inc.).

## Results

During the study period, there were 801 natural/unstimulated IVF cycles with oocyte aspiration performed as scheduled (Additional file 1: Figure S2). A premature LH surge (LH ≥ 20 mIU/ml) occurred in 39.32% (315/801) natural cycles, which is consistent with previous reports. Patterns of serum E2 in the preovulatory phase were divided into three groups, as shown in Table 1. Compared with the − 1 E2 level (the second to last), 403 (50.31%) participants with natural/unstimulated IVF cycles had preovulatory E2 level increases of 10% (Group A), 192 (23.97%) had preovulatory E2 levels within ±10% (Group B), and 206 (25.72%) demonstrated > 10% decreases in the preovulatory E2 level (Group C). There were no significant differences in age, BMI, serum basic FSH level, infertility duration, or antral follicle (AFC) number across the three groups (Table 1). Patients with a decrease in preovulatory E2 levels greater than 10% were more likely to have a spontaneous premature LH surge than patients with preovulatory E2 increase greater than 10% (53.40% vs. 30.02%, *P* < 0.05) (Table 2). Group C had significantly more natural/unstimulated IVF cycles cancelled because of premature ovulation compared with groups A and B (30.58% vs. 12.37%; 30.58% vs 4.71%, *P* < 0.001, respectively) (Table 2). To analyze whether this effect was similar in the cycles with premature LH surge, we examined the premature ovulation rate in cycles with a different trigger. The results showed that patients with E2 levels that decreased by more than 10% had the highest premature ovulation rate in cycles with premature LH surges and GnRHa/hCG triggered cycles (Additional file 1: Figure S3). Moreover, patients with E2 levels that decreased more than 10% and premature LH surges had higher premature ovulation rates than patients with E2 levels that decreased greater than 10% and those triggered by the hCG and GnRHa (35.55% vs 25.92, 35.55% vs 26.09%, *P* < 0.05, respectively) (Additional file 1: Figure S3). There was no significant difference in the premature ovulation rate between the cycles triggered with hCG and GnRHa.

**Table 1:** Characteristics of patients with different pre-ovulatory E2 level patterns

Characteristics	E2(ng/mL)			*P* Value
	Group A (> 10% increase) (*n* = 403)	Group B (±10% plateau) (*n* = 192)	Group C (> 10% decrease) (*n* = 206)	
Age of women (year)	39.87 ± 6.04	40.58 ± 5.82	40.27 ± 5.22	NS
BMI of women	21.74 ± 3.25	21.92 ± 2.57	21.51 ± 3.57	NS
Baseline FSH (mIU/mL)	9.54 ± 6.94	10.51 ± 8.88	9.30 ± 6.39	NS
AFC	1.78 ± 1.94	1.61 ± 1.71	1.76 ± 2.42	NS
Infertility duration(year)	3.93 ± 3.87	3.96 ± 4.22	3.79 ± 3.65	NS
Infertility causes, n (%)				
Tubal factor	268(66.5)	121(63.02)	132(64.08)	NS
Male factor	85(21.09)	44(22.92)	39(18.93)	NS
Endometriosis	65(16.13)	28(14.58)	44(21.36)	NS
Uterine factor	29(7.20)	15(7.81)	15(7.28)	NS
DOR	333(82.63)	167(86.98)	166(80.58)	NS

Data are presented as the means ±SD. *BMI* body mass index. a indicates group A versus group B,C

**Table 2:** Ovarian stimulation characteristics and outcomes of patients with different pre-ovulatory E2 level patterns

Characteristics	E2(ng/mL)			P Value
	Group A(> 10% increase) (n = 403)	Group B(±10% plateau) (n = 192)	Group C (> 10% decrease) (n = 206)	
No premature LH surge (HCG/Tiplilin trigger)	282(69.98%)	108(56.25%)	96(46.60%)	a
Premature LH surge	121(30.02%)	84(43.75%)	110(53.40%)	a
Pre-ovulatory P level	0.3 ± 0.48	0.5 ± 0.45	0.5 ± 0.33	NS
Number of oocytes retrieved	0.80 ± 0.496	0.76 ± 0.68	0.64 ± 0.55	b
Oocyte retrieval rate	75.99 ± 40.47	77.31 ± 38.64	77.28 ± 39.09	NS
Mature oocytes	0.70 ± 0.51	0.67 ± 0.67	0.55 ± 0.54	b
Mature oocytes rate (%)	89.00 ± 1.73	89.98 ± 2.75	87.70 ± 2.93	NS
Normal fertilization rate	89.62 ± 30.27	88.57 ± 31.11	84.82 ± 35.41	NS
Embryos frozen	0.40 ± 0.51	0.35 ± 0.51	0.27 ± 0.45	c
Top-quality embryos	0.32 ± 0.49	0.22 ± 0.43	0.16 ± 0.37	c
Premature ovulation	19(4.71%)	24(12.37%))	63(30.58%)	P < 0.001
IVF cycles without embryo frozen	241(59.8%)	122(63.5%)	142(68.9%)	c

Data are presented as the means ±SD. a indicates group A versus group B, C; b indicates group C versus group A, B; c indicates group C versus group A;

Group C had a lower mean oocyte yield compared to groups A and B (0.64 vs. 0.80, 0.64 vs. 0.76, *P* < 0.05 respectively). There were significantly less frozen embryos (0.27 vs. 0.4, P < 0.05) and more natural/unstimulated IVF cycles without embryos frozen (68.9% vs. 59.8%, P < 0.05) in group C compared with group A. However, there were no significant differences in the oocyte retrieval rate, mature oocyte rate, or normal fertilization rate.

Additionally, we performed a multivariable logistic regression to identify the individual factors associated with premature ovulation. Age, basal FSH level, trigger type (premature LH surge or hCG/GnRHa trigger), and preovulatory E2/− 1 E2 ratio were included as confounders. Multivariable logistic regression found that premature LH surge triggers and preovulatory E2/− 1 E2 ratios were significantly associated with premature ovulation (Table 3). These results indicated that preovulatory E2/− 1 E2 ratio and premature LH surge are two independent factors associated with premature ovulation.

**Table 3:** Multivariate logistic analysis of factors related to premature ovulation

Factor	*OR* [95% CI]	P value
Age – year	0.990(0.949–1.032)	0.637
Baseline FSH (mIU/mL)	1.016(0.983–1.049)	0.353
Premature LH surge	3.157(1.983–5.025)	< 0.001
Pre-ovulatory E2/−1E2 ratio	23.283 (10.348–52.389)	< 0.001

**Fig. 1:**
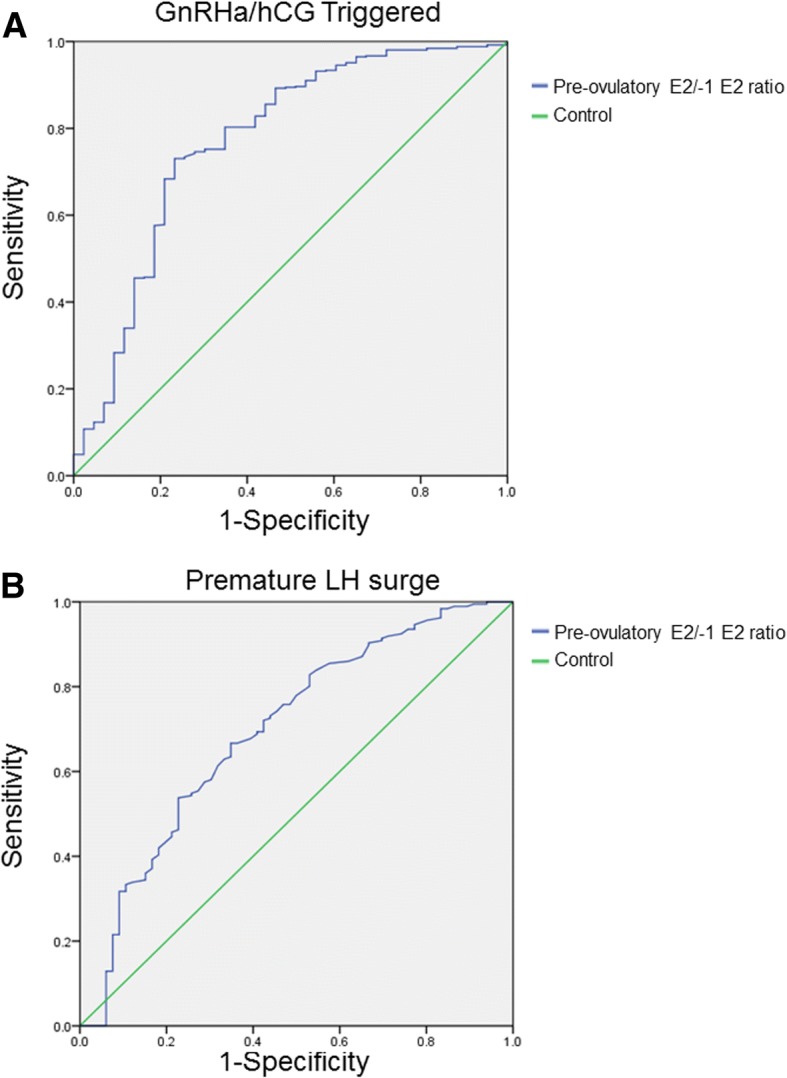
Receiver operation curve (ROC) analyses of premature E2/−1E2 ratios in the unstimulated IVF cycles (A) and in the cycles with premature LH surge (B). The cut-off value was 0.84 for premature E2/−1E2 ratios in unstimulated IVF cycles and 0.77 for premature E2/−1E2 ratios in cycles with premature LH surge

To analyze the accuracy of using preovulatory E2 decreases to predict premature ovulation, receiver operating curve analysis was performed in the cycles with premature LH surge and hCG/GnRHa triggered cycles. The results revealed that preovulatory E2/− 1 E2 ratio was a valuable marker for identifying premature ovulation, with an AUC (area under the receiver operating curve) of 0.772 in the hCG/GnRHa triggered cycles and AUC of 0.708 in the cycles with premature LH surge (Fig. 1). Therefore, a preovulatory E2 decrease > 23 and > 16% is a useful predictor of premature ovulation in cycles with premature LH surge and in hCG/GnRHa triggered cycles, respectively.

We predicted that emergency follicle aspiration might be an effective strategy to avoid premature ovulation. To investigate whether emergency OPU could decrease the rate of natural/unstimulated IVF cycle cancellation and increase natural cycle efficiency, we analyzed all natural/unstimulated cycles with preovulatory E2 level decreases greater than 23% who had OPU performed as scheduled or emergently. During the study period, there were 153 natural/unstimulated IVF cycles with preovulatory E2 decreases greater than 23% with OPU performed emergently (Table 4). The patients with preovulatory E2 decreases greater than 23% undergoing emergency OPU had a similar age, BMI, baseline FSH level, infertility duration, and infertility causes as those for whom follicle aspiration was performed as scheduled. There were only 2.61% (4/153) natural/unstimulated cycles with preovulatory E2 decreases who showed premature ovulation after emergency follicle aspiration was performed. This figure was significantly lower than that in the group with follicle aspiration performed as scheduled (2.61% vs. 43.82, *P* < 0.001). Emergency follicle aspiration did not increase the rate of empty oocyte syndrome and decreased the oocyte retrieval rate, mature oocyte rate, and normal fertilization rate (Table 4). Because fewer natural/unstimulated IVF cycles were cancelled when emergency OPU was performed, there were more oocytes and more frozen embryos than observed in the natural/unstimulated IVF cycles with preovulatory E2 levels decreasing more than 23% with follicle aspiration performed as scheduled (0.80 vs. 0.52, 0.40 vs. 0.24, *P* < 0.05, respectively). Furthermore, the natural/unstimulated IVF cycles without any embryos frozen decreased in the group with emergency follicle aspiration performed when the preovulatory E2 level decreased by more than 23% (59.5% vs. 75.2%, *P* = 0.007). These results showed that a preovulatory E2 decrease is a valuable marker for predicting premature ovulation and that emergency OPU is an effective strategy to decrease the rate of premature ovulation and cycle cancellation and increase natural/IVF cycle efficiency.

**Table 4:** Baseline characteristics, ovarian stimulation characteristics and outcomes of patients with pre-ovulatory E2 > 23% decreases with OPU performed as scheduled or emergently

Characteristic	Emergency OPU (*n* = 153)	OPU as scheduled (*n* = 117)	P Value
Age of women	40.09 ± 5.60	40.47 ± 5.45	0.409
BMI of women	21.28 ± 4.28	21.85 ± 2.59	0.179
Baseline FSH(mIU/mL)	10.83 ± 8.24	10.34 ± 4.42	0.435
Infertility duration	3.42 ± 2.65	3.21 ± 2.36	0.245
Infertility causes, n (%)			
Tubal factor	22(14.37)	19(16.24)	0.657
Male factor	31(20.26)	26(22.22)	0.696
Endometriosis	21(13.73)	20(17.09)	0.445
DOR	152(99.35)	114(97.44)	0.198
Uterine factor	0(0)	2(2.24)	0.105
hCG/Tiplilin trigger (%)	25(16.34)	30(25.64)	0.06
Premature LH surge	128(83.66)	87(74.35)	0.06
No. of oocytes retrieved	0.87 ± 0.41	0.58 ± 0.56	< 0.001
Oocytes retrieval rate (%)	76.71 ± 42.41	74.32 ± 43.98	0.443
Mature oocytes	0.80 ± 0.42	0.52 ± 0.55	< 0.001
Mature oocytes rate (%)	92.25 ± 26.85	88.89 ± 31.68	0.130
Fertilization rate (%)	84.68 ± 36.17	84.48 ± 36.52	0.946
Embryos frozen	0.40 ± 0.49	0.24 ± 0.43	< 0.001
Top-quality embryos	0.20 ± 0.40	0.17 ± 0.37	0.106
Premature ovulation rate	4(2.61)	48(41.03)	< 0.001
Empty Follicle syndrome	17(11.11)	11(9.40)	0.648
IVF cycle without embryo frozen	91(59.5%)	88(75.2%)	0.007
Implantation rate (%)	36.84(14/38)	27.27 (6/22)	0.148
Clinical pregnancy rate per transfer (%)	34.21(13/38)	22.73(5/22)	0.137
Miscarriage rate per transfer (%)	5.26(2/38)	9.09(2/22)	0.619
Live birth rate per transfer (%)	18.42(7/38)	9.09(2/22)	0.464
Ongoing pregnancy rate per transfer (%)	13.16(5/38)	9.09 (2/22)	1.000

Data are presented as the means ±SD, BMI: body mass index. DOR: Diminished ovarian reserve

In total, 66.67% (38/57) of embryos in the group with a preovulatory E2 decrease greater than 23% underwent emergency OPU, and 75.86% (22/29) of the embryos in the group with a preovulatory E2 decrease greater than 23% who underwent OPU as scheduled were thawed for FET (Table 4). The rate of implantation did not increase in the group with a preovulatory E2 decrease greater than 23% who underwent emergency OPU, nor did the clinical pregnancy and live birth rate or miscarriage rate increase (Table 4).

## Discussion

Although natural/unstimulated IVF cycle treatment has several advantages for ART, it is offered sparingly because monitoring these cycles is problematic due to the inability to prevent premature ovulation. Here, we found that decreases in preovulatory E2 levels are a valuable marker for predicting premature ovulation. Moreover, emergency follicle aspiration performed in natural/unstimulated cycles with a preovulatory E2 level decreases greater than 23% can decrease the rate of premature ovulation and avoid IVF cycle cancellation.

As early as 1986, Laufer et al. observed lower pregnancy rates per retrieval in women and a decrease in post-hCG E2 levels (13% vs. 19%). Cancellation of retrieval was advocated in patients who displayed a decrease in E2 levels [16]. Currently, cycle cancellation is widely accepted by physicians when premature LH levels surge and decreases in E2 levels are observed in natural cycles. In this study, we conducted a retrospective analysis of 801 natural/unstimulated cycles undergoing oocyte aspiration as scheduled. We found that the preovulatory E2 level was associated with fewer retrieved oocytes and cryopreserved embryos in natural/unstimulated IVF cycles. These results were consistent with a recent study on stimulated IVF cycles which showed that a decrease greater than 10% in E2 levels after hCG administration was associated with fewer retrieved oocytes and cryopreserved embryos compared to the group with increases in E2 levels greater than 10% [20]. We further found that preovulatory E2 decreases were associated with premature ovulation in natural/unstimulated IVF cycles. However, there were no significant difference in the rates of oocyte retrieval, oocyte maturation, and normal fertilization. Our results indicate that decreases in E2 levels were associated with premature ovulation, but did not negatively impact oocyte retrieval, maturation, or quality.

In natural cycles, decreases in E2 levels following an LH surge are due to reduced androgen production and aromatase activity because granulosa cell aromatase activity and follicular fluid aromatizable androgen to estrogen concentration ratio were lowest in preovulatory follicles [21]. However, LH surges that result in ovulation are extremely variable in configuration, amplitude, and duration [22, 23]. It is difficult to estimate the timing of follicle aspiration from LH levels because premature ovulation frequently occurs 24 h after LH increases > 20 mIU/ml. We predicted that the E2 decreases following surges in LH levels might be a marker for predicting premature ovulation. Consistent with this result, we found that decreases in E2 levels are a valuable marker for predicting premature ovulation. After emergency follicle aspiration, mature oocytes can be retrieved, and high-quality embryos can be developed using the retrieved oocytes. Moreover, there were no significant differences in the rates of oocyte retrieval and oocyte maturation. These results indicated that emergency follicle aspiration was the right choice for patients who experienced decreases in E2 levels. In this study, we observed more natural IVF cycles with spontaneous LH surges in Group C (E2 level > 10% decrease) than in Group A (E2 level > 10% increase), which might be due to the variation of spontaneous LH surges in terms of configuration, amplitude and duration.

Progesterone has been suggested as a marker for monitoring natural IVF cycles [10]. In prior work, high serum progesterone concentrations on the day of HCG administration were found negatively impact IVF outcomes [24, 25]. In this study, we also constructed a receiver operation characteristic curve to identify the predictive value for premature ovulation in natural IVF cycles. Results showed that preovulatory progesterone levels had poor predictive value for the risk of premature ovulation (data not shown).

Inhibin A begins to rise in the late follicular phase, reaching peak levels at mid-cycle and in the mid-luteal phase [26]. Patterns of inhibin A vary with maturational stage of the follicle [27, 28]. In this study, we did not measure inhibin A levels. Future studies should further examine the correlation between inhibin A levels, E2 levels, and premature ovulation.

The majority of the patients in this study were over 40 years of age and/or diagnosed as DOR. For these patients, successful oocyte retrieval is important for their therapy, and cycle cancellation might make them permanently lose the chance for pregnancy. We found that a preovulatory E2 decrease is a useful marker for predicting premature ovulation and that emergency OPU can efficiently decrease the rate of premature ovulation and avoid cycle cancellation. Moreover, emergency OPU did not decrease the rate of mature oocytes, the oocyte retrieval rate, or the implantation rate, nor did it increase the miscarriage rate. This strategy increased the chance of pregnancy, especially for older patients and patients with DOR.

## Conclusions

In conclusion, this is the first study demonstrating that closely monitoring serum E2 levels can predict premature ovulation and that emergency follicle aspiration can help patients avoid premature ovulation and cancellation of natural/unstimulated IVF cycles.

## Additional file

Additional file 1: Supplementary information. **Figure S1.** The process of natural/unstimulated cycles. OPU: ovum pick-up. **Figure S2.** Flow chart of patients for the study. OPU: ovum pick-up; n: number. **Figure S3.** The premature ovulation rate in the group with E2>10% increase, E2± 10% plateau, and E2>10%decrease in the cycles triggered with hCG, GnRHa (Triplilin), and premature LH surge respectively. * indicate P<0.05. (DOCX 422 kb)
