# Supplementary material for: Decrease in preovulatory serum estradiol is a valuable marker for predicting premature ovulation in natural/unstimulated in vitro fertilization cycle
Source: J Ovarian Res. 2018 Nov 21;11:96. doi: 10.1186/s13048-018-0469-x (PMC6247609; doi:10.1186/s13048-018-0469-x)
Supplement: Supplementary file 1 — Supplementary information. Figure S1. The process of natural/unstimulated cycles. OPU: ovum pick-up. Figure S2. Flow chart of patients for the study. OPU: ovum pick-up; n: number. Figure S3. The premature ovulation rate in the group with E2>10% increase, E2± 10% plateau, and E2>10%decrease in the cycles triggered with hCG, GnRHa (Triplilin), and premature LH surge respectively. * indicate P<0.05. (DOCX 422 kb) [file 13048_2018_469_MOESM1_ESM.docx]

**Supporting information**

**Running title: The effect of preovulatory E2 decrease in natural/unstimulated IVF cycles**

**Decrease in preovulatory serum estradiol is a valuable marker for predicting premature ovulation in natural/unstimulated in vitro fertilization cycle**

Xuefeng Lu^1#^, Shuzin Khor^#^, Qianqian Zhu^1#^, Lihua Sun^2^, Yun Wang^1^, Qiuju Chen^1^, Ling Wu^1^, Yonglun Fu^1^, Hui Tian^2^, Qifeng Lyu^1^, Renfei Cai^1*^, and Yanping Kuang ^1*^

^1^ Department of Assisted Reproduction, Shanghai Ninth People's Hospital, Shanghai Jiaotong University School of Medicine, 639 Zhizaoju Rd, Shanghai 200001, China

^2^ Department of Assisted Reproduction, Shanghai East Hospital, Shanghai Tongji University School of Medicine, Shanghai 200120, China

^#^these authors contribute equally to this work

^*^Corresponding author: Yanping Kuang and Renfei Cai

Department of Assisted Reproduction, Shanghai Ninth People's Hospital, Shanghai Jiaotong University School of Medicine, 639 Zhizaoju Rd, Shanghai 200001, China.

E-mail: [kuangyanp@126.com](mailto:kuangyanp@126.com) and cairenfei070@sina.com

Tel: +86-21-23271699-5539; Fax: +86-21-53078108

**There is no conflict of interest to declare.**


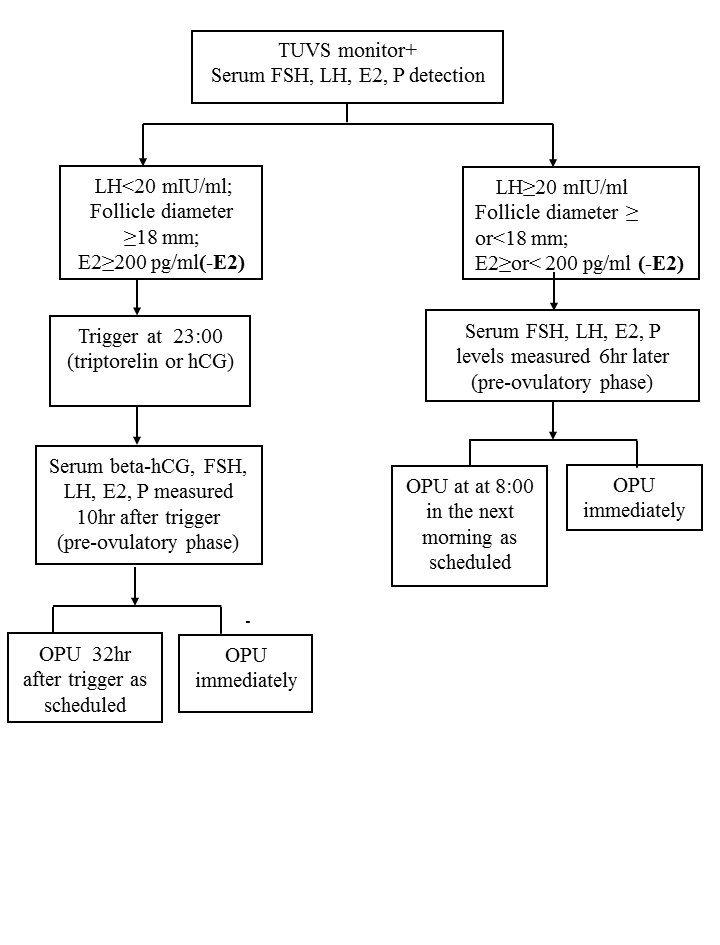


S1 Fig. The process of natural/unstimulated cycles. OPU: ovum pick-up.


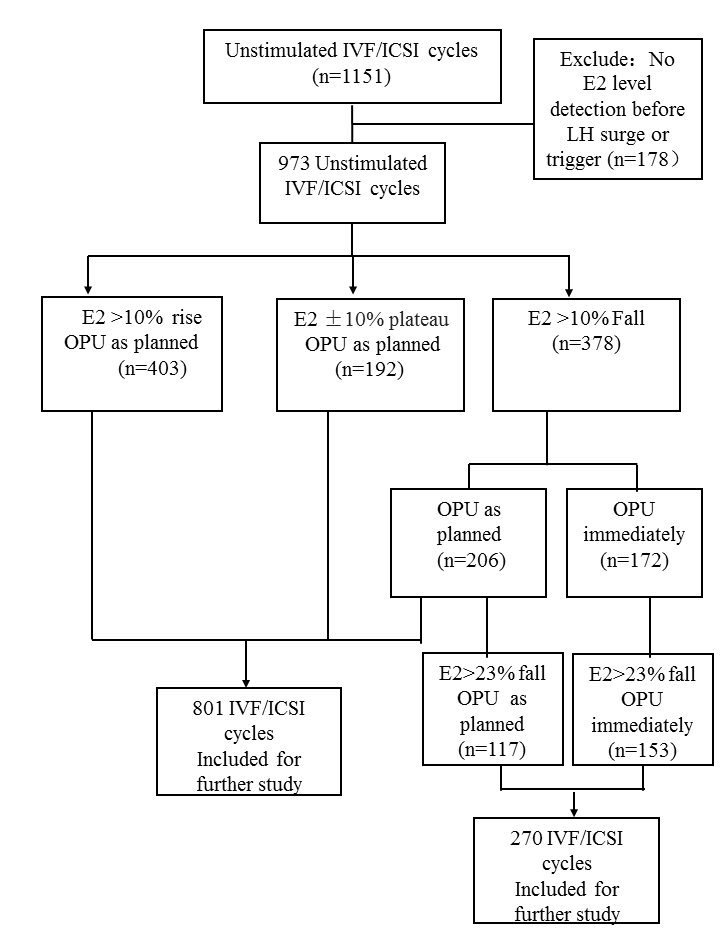


S2 Fig. Flow chart of patients for the study. OPU: ovum pick-up; n: number.


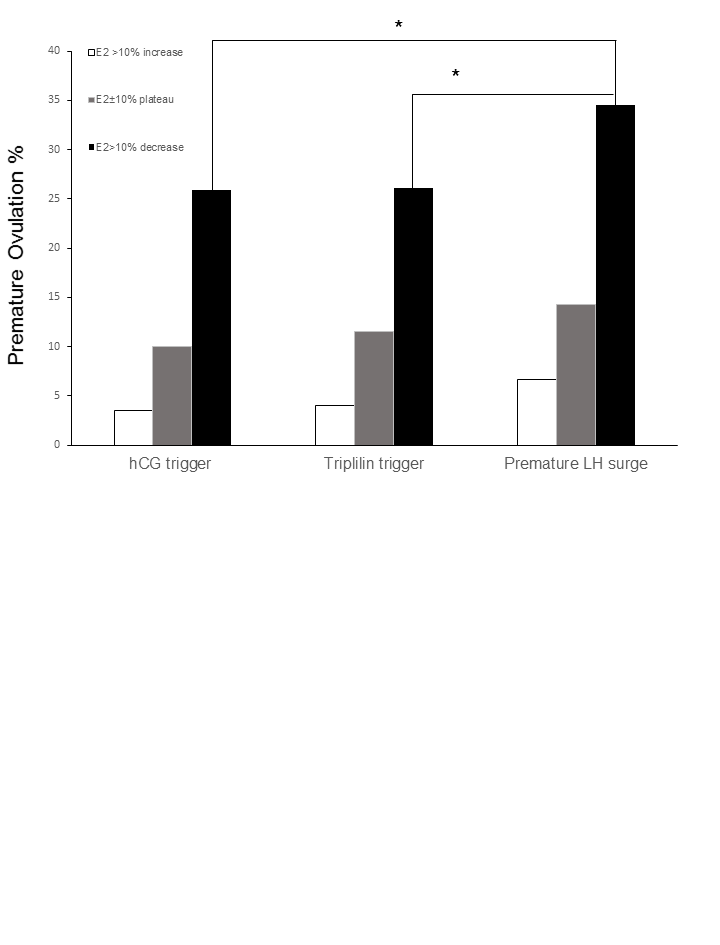


S3 Fig. The premature ovulation rate in the group with E2>10% increase, E2± 10% plateau, and E2>10%decrease in the cycles triggered with hCG, GnRHa (Triplilin), and premature LH surge respectively. * indicate P<0.05.
